# Saudi women's perspectives on postpartum depression

**DOI:** 10.3389/fgwh.2024.1326130

**Published:** 2024-03-14

**Authors:** Asmahan Alsulami, Abeer Orabi, Shahrazad Timraz

**Affiliations:** ^1^ Department of Labour and Delivery, King Abdulaziz University Hospital, Jeddah, Saudi Arabia; ^2^ College of Nursing, King Saud bin Abdulaziz University for Health Sciences, Jeddah, Saudi Arabia; ^3^ Department of Nursing, King Abdullah International Medical Research Center, Jeddah, Saudi Arabia

**Keywords:** postpartum depression, knowledge, attitude, perspectives, Saudi women

## Abstract

**Introduction:**

Postpartum depression (PPD) is a serious disorder that affects women worldwide, making it a crucial public health concern and one of the most prevalent childbirth complications. Assessing the perspectives of Saudi women towards PPD is essential for addressing the issue and developing effective solutions. This study aimed to assess the knowledge and attitude of Saudi women about PPD in King Abdulaziz Medical City, Western Region.

**Methods:**

This study employed a descriptive cross-sectional research design and included 226 women recruited through convenience sampling at the Obstetrics and Gynaecology Outpatient Department in King Abdul-Aziz Medical City, Jeddah. Data were collected using a structured questionnaire with 3 main sections: socio-demographic characteristics, knowledge regarding risk factors and symptoms of PPD, and attitude about PPD.

**Results:**

The mean score (SD) of the knowledge scale was 31.8 (6.2) with 53.5% of participants having a good level of knowledge about PPD. The mean score (SD) of the attitude scale was 27.1 (2.8) with 47.3% of participants having a positive attitude towards PPD. Significant differences were identified in the knowledge scale, where women who were postgraduates, working in the health sector and unpregnant scored higher in the knowledge scale compared to others (*P* = .008, *P* = .02, & *P* = .008, respectively).

**Discussion:**

The findings of this study indicate that women's knowledge about PPD was generally good; however, many of them held negative attitudes towards PPD. These findings underscore the importance of proper education to improve mental health literacy and address prejudices and negative stereotypes related to PPD among Saudi women.

## 1 Introduction

Postpartum depression (PPD) is a complex condition affecting new mothers, marked by symptoms of depression typically starting around the fourth week after delivery but can occur anytime within the first year postpartum (1). The Diagnostic and Statistical Manual of Mental Disorders (DSM-5) defines PPD as a major depressive episode that begins during pregnancy or within four weeks after delivery. Diagnosis requires the presence of at least five depressive symptoms that occur almost daily for at least two weeks, significantly changing the individual's previous routine. These symptoms include depressed mood, lack of interest or pleasure, sleep disturbances, agitation or psychomotor retardation, feelings of guilt or worthlessness, fatigue, suicidal ideation or attempts, recurrent thoughts of death, weight or appetite changes, and difficulty concentrating or making decisions (2, 3). Early identification and management are critical to prevent the evolution into more severe mood disorders, emphasizing the importance of supporting the emotional and mental health shifts women experience during this period (4).

PPD has significant long-term effects on individuals and families, highlighting its importance as a public health issue (5). Untreated, PPD can lead to chronic mental health issues, suicidal tendencies, and adverse impacts on infant development and behavior, such as poor cognitive development, anxiety, emotional and mood disorders, behavioral problems, communication difficulties, and insecure attachments (6–8). These impacts can extend to the broader family unit, potentially leading to a diminished quality of life and psychological distress among partners (6), underlining the need for comprehensive support and intervention strategies (9). Depression rates among fathers range from 2% to 25% during their partner's pregnancy or the first year after delivery, increasing to 50% when the woman also suffers from PPD (10).

The global prevalence of PPD varies, as research from different regions indicates. Kaya and Çiğdem (11) observed its rising prevalence in developed countries, while Wang et al. (12) estimated a global prevalence of 17.22%. In Arabic countries, prevalence rates range from 8% to 40%, with studies in Saudi Arabia documenting high prevalence rates (13–16). The causes of PPD are multifaceted, including life stressors, lack of social support, and domestic violence. Systemic and sociopolitical factors significantly influence PPD risk, such as the quality of social support networks, healthcare access, and cultural attitudes towards motherhood and mental health (17, 18). Social isolation, inadequate healthcare access, and societal pressures or stigmas related to motherhood can exacerbate PPD risks (18, 19).

Given the considerable impact of PPD on individuals, families, and communities, understanding women's knowledge and attitudes towards this condition is paramount, especially in regions like Saudi Arabia. Enhancing women's ability to recognize symptoms and seek help is essential, considering the significant influence of cultural norms and social support systems on mental health outcomes. This focus on knowledge and attitude is crucial for developing targeted educational and intervention programs to address mental health stigma and improve support for new mothers during the postpartum period.

## 2 Materials and methods

### 2.1 Study design

A descriptive cross-sectional research design was employed to assess the knowledge and attitudes of Saudi women about PPD.

### 2.2 Study setting, population, and sampling

Women attending the Obstetrics and Gynecology Outpatient Department at King Abdul-Aziz Medical City in Jeddah were conveniently recruited for the current study. The inclusion criteria were Saudi nationality, being married, age 18 years or older, and a willingness to participate in the study. The estimated sample size was 226, determined using Raosoft software. Input data included a population size of 1,348 within the three-month period of data collection, as per Obstetrics and Gynecology Outpatient Department Statistics (2021). A margin of error of 5.0% and a confidence level of 90% were considered.

### 2.3 Data collection

A structured questionnaire was used for data collection, originally developed by Chongpanish et al. (20) and previously used by Poreddi et al. (21). The questionnaire consisted of 3 main sections: Section (1) gathered participants' socio-demographic characteristics, including age, level of education, occupation, obstetric history (gravidity and parity), and medical history. Section (2) collected information on women's knowledge regarding risk factors (15 items) and signs and symptoms of PPD (8 items). Responses were on a 3-point Likert scale: true (2), don't know (1), and false (0). The total score for this section ranged from 0 to 46, with a higher score indicating better knowledge of PPD. Section (3) assessed attitudes towards PPD through 11 statements, including 9 negatively worded items. Participants were asked to respond based on their level of agreement with each statement using a 3-point Likert scale: agree (3), neutral (2), and disagree (1). The total score ranged from 11 to 33, with higher scores indicating more positive attitudes towards PPD.

The questionnaire's validity was assessed by a panel of experts in nursing and obstetrics, who rated each item in terms of relevance to the construct and clarity on a 4-point ordinal scale. The item-level Content Validity Index (I-CVI) was calculated as the number of experts who rated an item as 3 or 4, divided by the total number of experts. Subsequently, the Scale-level Content Validity Index (S-CVI/Ave) was computed, yielding a value of 0.91, which is considered acceptable. The reliability of the instrument was ensured through a pilot study. The results showed a Cronbach's Alpha of 0.668 for the knowledge scale and 0.701 for the attitude scale, both considered acceptable.

Data was collected through face-to-face interviews using an Arabic version of the questionnaire. The researcher explained the study's purpose, obtained informed consent from each participant, and conducted the interviews in the waiting area of the Obstetrics and Gynecology Outpatient Department. The data collection was carried out over a three-month period in 2022. A pilot study was conducted with 45 women (20% of the study participants) who met the inclusion criteria to assess questionnaire completeness, accuracy, and item clarity, and no modifications to the translated scale were needed.

### 2.4 Data analysis

The collected data were coded, entered, and analyzed using the Statistical Package for Social Sciences (SPSS) version 29. Descriptive statistics, including frequencies, percentages, means, and standard deviations, were used for data analysis. Inferential statistics, such as *t*-tests and ANOVA, were employed to examine associations and the significance of comparisons between variables. Pearson correlation was used to assess the relationship between variables, with a significance level set at *P* ≤ 0.05.

### 2.5 Ethical consideration

The study was conducted after obtaining approval from the Institutional Review Board (IRB) at King Abdulaziz Medical City, Western Region, and King Abdullah International Medical Research Center (KAIMRC), Jeddah, KSA. The researcher obtained written consent from each eligible participant after explaining the study's aim and nature. Participation was voluntary, and participants had the right to withdraw from the study at any time without any obligations. Confidentiality was maintained, with no identifiers collected, and all data was securely stored.

## 3 Results

### 3.1 Description of the participants

The present study included 226 women, with a mean age (SD) of 32.3 (7.5) years. More than half of the participants were university graduates (53.1%), and the majority were married (98.7%). Additionally, more than three-quarters of the participants were housewives (78.8%). The mean values (SD) for gravidity, parity, and abortion were 3.3 (2.5), 2.2 (2.0), and 0.6 (0.5), respectively. More than half of the participants were pregnant (52.7%), and 62.2% of their pregnancies were planned. The mean gestational weeks (SD) were 29.0 (9.5). Furthermore, 13.3% of the participants had chronic health conditions, with hypothyroidism accounting for 43.3% (Table 1).

**Table 1:** Socio-demographic characteristics of the participants.

Item	*N* = 226
Age: Mean (SD)	32.3 (7.5)
Educational level	
Elementary school	10 (4.4)
Intermediate school	14 (6.2)
High school	73 (32.3)
University graduated	120 (53.1)
Postgraduate	9 (4.0)
Marital status	
Married	223 (98.7)
Divorced	2 (0.9)
Widow	1 (0.4)
Occupation	
Housewife	178 (78.8)
Working in the health sector	15 (6.6)
Working outside the health sector	33 (14.6)
Obstetric history	
Gravidity: Mean (SD)	3.3 (2.5)
Parity: Mean (SD)	2.2 (2.0)
Abortion: Mean (SD)	0.6 (0.5)
Pregnancy status	
Unpregnant	107 (47.3)
Planned pregnancy	74 (62.2)
Unplanned pregnancy	45 (37.8)
Weeks of gestation: Mean (SD)	29.0 (9.5)
Chronic medical diseases	
No	196 (86.7)
DM	7 (23.3)
HTN	5 (16.7)
Hypothyroidism	13 (43.3)
Lupus	1 (3.3)
Asthma	3 (10.0)
Multiple sclerosis	1 (3.3)

### 3.2 Knowledge about PPD

In terms of the risk factors associated with PPD, 76.5%, 73.5%, 73.0%, and 71.2% of the participants reported increased work pressure or stress, domestic violence, lack of family support, and husband's substance abuse, respectively. Additionally, 62.4% and 61.9% of the participants identified poverty/financial difficulties and crises as contributing factors to the development of PPD. Moreover, 53.1% of the participants outlined that being a single woman (divorced or widowed) is a risk factor for PPD. In contrast, 52.2%, 49.9%, 42.9%, and 36.3%, respectively, of the participants believed that genetic/hereditary factors, poor maternal education, disappointment with the gender of the newborn, and the young age of the woman are risk factors for PPD.

Many of the participants identified feelings of sadness, fatigue/weakness, sleeping problems, and a lack of bonding with the newborn as symptoms of PPD, with percentages of 89.4%, 80.5%, 76.1%, and 70.8%, respectively. The mean score (SD) for the knowledge scale was 31.8 (6.2). Using the mean as a cutoff point, the scale was categorized as knowledgeable (31.8 and above) and not knowledgeable (less than 31.8). The percentage of knowledgeable participants was 53.5%, while 46.5% of the participants were not knowledgeable (Table 2).

**Table 2:** Women's knowledge about PPD.

Item				True	I don't know	False
Risk factors of PPD						
1. Genetic/hereditary				30 (13.3)	78 (34.5)	118 (52.2)
2. Crisis situation				140 (61.9)	59 (26.1)	27 (11.9)
3. History of depression				102 (45.1)	81 (35.8)	43 (19.0)
4. Lack of family support				165 (73.0)	19 (8.4)	42 (18.6)
5. Ghost possessed/doing sin/Black magic				34 (15.0)	76 (33.6)	116 (51.3)
6. Health problem/sickness of baby				165 (73.0)	31 (13.7)	30 (13.3)
7. Domestic violence				166 (73.5)	29 (12.8)	31 (13.7)
8. Age (below 20 years)				85 (37.6)	59 (26.7)	82 (36.3)
9. Disappointment with gender of the baby				81 (35.8)	48 (21.2)	97 (42.9)
10. Poverty/financial difficulties				141 (62.4)	36 (15.9)	49 (21.7)
11. Poor education of the woman				77 (34.1)	37 (16.4)	112 (49.6)
12. Increased work pressure or stress				173 (76.5)	31 (13.7)	22 (9.7)
13. Physical weakness				84 (37.2)	56 (24.8)	86 (38.1)
14. Substance abuse among husbands				161 (71.2)	34 (15.0)	31 (13.7)
15. Single woman (Divorced or Widow)				120 (53.1)	63 (27.9)	43 (19.0)
Symptoms of PPD						
1. Feeling sad/miserable				202 (89.4)	16 (7.1)	8 (3.5)
2. Lack of bonding or worry about bonding with baby				160 (70.8)	40 (17.7)	26 (11.5)
3. Not interested in doing household chores				124 (54.9)	42 (18.6)	60 (26.5)
4. Not interested in talking with others				153 (67.7)	39 (17.3)	34 (15.0)
5. Feeling of fatigue/weakness				182 (80.5)	24 (10.6)	20 (8.8)
6. Sleeping problems				172 (76.1)	34 (15.0)	20 (8.8)
7. Irritability				127 (56.2)	77 (34.1)	22 (9.7)
8. Death wishes				145 (64.2)	47 (20.8)	34 (15.0)
Scale	Min	Max	Mean	Median	SD	IQR
Knowledge	13	44	31.8	32.0	6.1	8.3

### 3.3 Attitude toward PPD

Nearly half of the participants 55.3% and 44.7% respectively, agreed that PPD is common and increases the risk of committing suicide. Furthermore, 14.2%, 16.8%, 28.8%, and 36.3% of the participants agreed that a woman with PPD should be separated from her baby, feel shame, and hide her condition, be unable to take care of her own children, and incapable of making any decisions. On the other hand, majority of the participants disagreed with the idea that women with PPD should stay at home (90.3%) and cannot be good women (88.9%). Moreover, 77.4% and 76.5% of the participants respectively disagreed that PPD does not require any treatment and that women who have PPD should not have another child. The mean score (SD) of the attitude scale was 27.1 (2.8). The percentage of participants with a positive attitude (score of more than 27) was 47.3%, compared to 52.7% with a negative attitude (Table 3).

**Table 3:** Women's attitude toward PPD.

Item				Agree	Neutral	Disagree
1. I feel shame and do not tell anyone that I have PPD.				38 (16.8)	60 (26.5)	128 (56.6)
2. Postpartum women who have PPD cannot be good women.				7 (3.1)	18 (8.0)	201 (88.9)
3. Postpartum women who have PPD should stay at home.				7 (3.1)	15 (6.6)	204 (90.3)
4. Postpartum women who have PPD cannot take care of her own children.				65 (28.8)	87 (38.5)	74 (32.7)
5. Postpartum women who have PPD cannot make decisions at all.				82 (36.3)	67 (29.6)	77 (34.1)
6. Postpartum women who have PPD should not have another child.				18 (8.0)	35 (15.5)	173 (76.5)
7. If a woman develops PPD, woman and baby should be separated.				32 (14.2)	74 (32.7)	120 (53.1)
8. Postpartum depression does not require any treatment.				21 (9.3)	30 (13.3)	175 (77.4)
9. Postpartum depression is common among women				125 (55.3)	69 (30.5)	32 (14.2)
10. Breast feeding should be stopped if a woman develops PPD.				20 (8.8)	57 (25.2)	149 (65.9)
11. Woman with PPD have a high risk of committing suicide.				101 (44.7)	83 (36.7)	42 (18.6)
Scale	Min	Max	Mean	Median	SD	IQR
Attitude	18	33	27.1	27.0	2.8	4.0

In terms of differences in knowledge level of women based on their sociodemographic characteristics, significant disparities were observed concerning educational level, occupation, and pregnancy status. As women who were postgraduates, working in the health sector, and unpregnant exhibited significantly higher scores in the knowledge scale when compared to other women (*P* = .008, *P* = .020, & *P* = .008, respectively) (Table 4). The results of the correlation analysis between the quantitative basic characteristics and knowledge scale revealed a significant and positive correlation (*P* = .04) between gestational weeks and knowledge about PPD (Table 5). However, there were no significant differences between the basic characteristics of women and their attitude toward PPD (Tables 6, 7).

**Table 4:** Socio-demographic characteristics of participants by the knowledge scale.

Item	Scale mean (SD)	*P*-value
Educational level		
Elementary school	32.7 (6.4)	.008*
Intermediate school	29.6 (6.7)	
High school	30.7 (6.4)	
University graduated	32.3 (5.6)	
Postgraduate^a^	37.5 (4.4)	
Occupation		
Housewife	31.5 (6.2)	.02*
Working in the health sector^a^	36.1 (6.5)	
Working outside the health sector	31.8 (4.4)	
Pregnancy status		
Yes	30.8 (6.3)	.008*
No^a^	32.9 (5.7)	
If currently pregnant, this pregnancy was planned		
Yes	31.1 (6.3)	.49
No	30.3 (6.3)	
Chronic medical diseases		
Yes	30.8 (6.1)	.32
No	32.0 (6.1)	

^a^ Significant category.

* Significant difference.

**Table 5:** Correlation between knowledge scale and quantitative basic characteristics.

Item	Correlation	*P*-value
Age	−0.023	.77
Gravidity	−0.084	.20
Parity	−0.031	.57
Abortion	−0.072	.27
Gestation weeks	0.174	.04*

* Significant correlation.

**Table 6:** Socio-demographic characteristics of participants by attitude scale.

Item	Scale mean (SD)	*P*-value
Educational level		
Elementary school	27.7 (2.8)	.35
Intermediate school	26.4 (4.3)	
High school	27.5 (2.7)	
University graduated	27.1 (2.7)	
Postgraduate	25.9 (2.2)	
Occupation		
Housewife	27.3 (2.8)	.23
Working in the health sector	26.7 (2.7)	
Working outside the health sector	26.5 (2.7)	
Pregnancy status		
Yes	27.1 (2.9)	.87
No	27.2 (2.7)	
If currently pregnant, this pregnancy was planned		
Yes	27.0 (2.8)	.75
No	27.2 (3.0)	
Chronic medical diseases		
Yes	26.8 (3.8)	.51
No	27.2 (2.6)	

**Table 7:** Correlation between attitude scale and quantitative basic characteristics.

Item	Correlation	*P*-value
Age	−0.028	.67
Gravidity	0.059	.37
Parity	0.024	.72
Abortion	0.103	.12
Gestation weeks	0.028	.76

## 4 Discussion

In the present study, nearly two-thirds of the participants believed that domestic violence (73.5%), lack of family support (73.0%), husband's substance abuse (71.2%), and poverty (62.4%) were risk factors for PPD. These findings were supported by previous research conducted by Poreddi et al. (21) to explore the knowledge and attitudes of family members of postpartum women about PPD. The study found that domestic violence, substance abuse among husbands, lack of family support, and poverty were risk factors for PPD. Moreover, domestic violence was found to have a clear association with PPD among women in Asia in a systematic review conducted by Koirala and Chuemchit (22). Regarding the effect of family support and income on PPD, a study aimed at examining the risk factors of PPD revealed that PPD was less likely to be experienced by women who had strong family support than those with weak family support. Additionally, postpartum women with high family income are more able to meet their needs compared to postpartum women with poor family income who struggle to meet their daily demands, which in turn impacts the incidence of PPD (23).

Over half of the participants (53.1%) in this study reported that being a single woman (divorced or widowed) was a risk factor for PPD. A similar finding was reported in a study conducted by Obioha et al. (24) to evaluate the knowledge, attitude, and prevalence of PPD among postnatal women attending immunization clinics in three Primary Health Centers in Nigeria. It was found that marital status was a predictor of PPD, with unmarried women being 4.92 times more likely to experience PPD than married women. More than half of the participants in this study (52.2%) were unaware that genetic/hereditary factors were a risk factor for PPD. However, a study by Afolayan et al. (25) assessing the knowledge of PPD and its associated risk factors among nurse-midwives in Bayelsa State, Nigeria, found that 97.4% of the respondents attributed genetic makeup to the development of PPD. These findings may be attributed to the theoretical background of the participants regarding PPD. Additionally, McEvoy et al. (26) provided substantial support for the genetic basis of PPD in their qualitative literature review exploring the genetic basis for PPD. Only 37.6% of the participants identified the age of the woman (below 20 years) as a risk factor for developing PPD. Consistently, Agnafors et al. (27), who conducted a study investigating the effect of young maternal age on maternal mental health, found that young maternal age was associated with symptoms of PPD. Of women aged 20 or younger at childbirth, 23.0% reported symptoms of PPD, compared to 11.6% of women aged 21 and older.

Most of the participants were able to recognize that feeling sad (89.4%), fatigue/weakness (80.5%), sleeping problems (76.1%), and lack of bonding with the newborn (70.8%) were symptoms of PPD. These findings were in line with previous studies. In Branquinho et al. (28) study aiming to characterize the Portuguese general population's knowledge and attitudes about PPD and analyze its sociodemographic and clinical correlates, findings revealed that 57.3% of the participants identified severe sadness and irritability as symptoms of PPD, and 81.5% agreed that PPD affected women's sleep. Additionally, in a study conducted by Poreddi et al. (21), the majority of the participants were able to identify the symptoms of PPD, such as feeling sad (98%), sleeping problems (95.5%), lack of bonding with the baby (81.2%), and fatigue/weakness (87.1%).

Regarding the participants' attitudes toward PPD, over half of the participants in this study believed that PPD is common among women. In the same perspective, a systematic review and meta-analysis conducted by Wang et al. (12) to review the global epidemiology of PPD found that the estimated PPD prevalence was 17.22%.

Nationally, research in Saudi Arabia has shown incidence rates of 38.50% and 20.9% among Saudi women in Riyadh and Jeddah, respectively (14, 16). Also, 44.7% of the participants agreed that women who have PPD are at a higher risk of committing suicide. In this regard, the prevalence of suicidal ideation and attempted suicide was equal to 12% and 4%, respectively, based on a systematic review and meta-analysis conducted by Amiri and Behnezhad (29) to investigate the prevalence of postpartum suicidal ideation, suicide attempts, and suicide mortality.

Moreover, most of the participants disagreed that women who have PPD should stay at home (90.3%), cannot be good women (88.9%), and should not have more children (76.5%). In contrast, a prior study conducted by Poreddi et al. (21) reported that most of the participants agreed that women who have PPD cannot be good women (70.8%), should stay at home (70.3%), and should not have another child (61.9%). The disparity in findings between this study and Poreddi et al. (21) study may be due to differences in cultural settings. Findings have shown that most of the participants (77.4%) agreed on the importance and benefits of the treatment of PPD. Likewise, the World Health Organization (WHO) emphasized the importance of mental health support in the postnatal period, as well as the necessity of screening for PPD, with referral and treatment (30).

The study results suggest that women with high educational backgrounds (postgraduate) were found to have higher knowledge about PPD compared to women with lower educational levels. Contrarily, a descriptive quantitative study conducted by Abazie and Usoro (31) to assess the knowledge of PPD among women attending immunization clinics in Mushin, Nigeria, showed no significant relationship between education level and knowledge of PPD. This study found that women working in the health sector were more knowledgeable about PPD compared to others. On the contrary, a study conducted by Elshatarat et al. (32) addressed perinatal nurses' and midwives' knowledge about the assessment and management of PPD in Saudi Arabia. Results showed that nurses and midwives lacked knowledge about various aspects of PPD, including its definition, prevalence, symptoms, risk factors, screening tools, and treatment.

The strengths of this study encompass its rigorous methodological approach, including the use of a descriptive cross-sectional design and a structured questionnaire to assess knowledge and attitudes towards PPD among 226 Saudi women. Its comprehensive data collection, validated through expert panel assessment, provided a detailed understanding of participants' perspectives. The significant findings related to knowledge levels and attitudes towards PPD underscore the importance of targeted educational interventions. Additionally, the study's identification of sociodemographic factors influencing PPD awareness offers valuable insights for tailoring future public health strategies to improve mental health literacy among Saudi women.

However, this study has certain limitations. First, the sample was collected from a single tertiary hospital, which may restrict the generalizability of the findings. Moreover, the current study used non-probability sampling, which might introduce bias when recruiting the participants.

Future research studies that employ probability sampling methods are needed to achieve broader and more diverse samples and ensure a comprehensive representation of sociodemographic backgrounds. There is a critical need for educational programs that delve into the symptoms, origins, consequences, and treatments of PPD. These programs must be multifaceted, incorporating digital platforms, community outreach, and healthcare settings to ensure wide-reaching impact and promote early detection and intervention. Collaborative efforts among healthcare professionals, including nurses, midwives, and obstetricians, are essential for integrating physical and mental health support during the postnatal period. Finally, policymakers and healthcare administrators should prioritize educational initiatives within obstetric care, ensuring all women are informed about PPD to foster improved mental health support infrastructure. This holistic strategy not only aims to better individual outcomes but also seeks to shift societal perspectives towards a more understanding and proactive approach to managing PPD.

## 5 Conclusion

The findings of the present study on women's awareness of (PPD) showed that more than half of the participants had a good level of knowledge about PPD. Nonetheless, a larger portion of them had negative attitudes toward PPD. These findings underline the importance of understanding the disorder in the parenthood context. It shows that while many women are informed about PPD, negative attitudes persist. This highlights the significance of tailored educational programs facilitated by healthcare professionals, including obstetricians, midwives, and mental health experts. These programs can utilize online platforms to extend their reach, employing websites, social media, and virtual workshops to disseminate information widely and engage with a larger audience effectively. Additionally, integrating PPD education into prenatal and postnatal care plans ensures direct communication of information. Community-based interventions, such as support groups led by trained facilitators, can foster a more positive outlook toward PPD and encourage open discussions to address prejudices and stereotypes prevalent among Saudi women.

## Data Availability

The raw data supporting the conclusions of this article will be made available by the authors, without undue reservation.
